# Editorial: Enhanced biological mechanism study, drug discovery and individualized medicine with single-cell multiomics data and integrative analysis

**DOI:** 10.3389/fgene.2023.1236126

**Published:** 2023-06-26

**Authors:** Xinying Zhang, Panwen Wang, Jing Qin

**Affiliations:** ^1^ School of Pharmaceutical Sciences (Shenzhen), Shenzhen Campus of Sun Yat-sen University, Shenzhen, China; ^2^Department of Quantitative Health Sciences, Mayo Clinic Arizona, Scottsdale, AZ, United States

**Keywords:** individualized medicine, multi-omics, prognostic biomarker, therapeutic targets, computational approach

Individualized medicine, also known as personalized medicine or precision medicine, is a field in which medical decisions concerning disease prevention, diagnosis, and treatment are tailored to individual patients based on their genetic information (König et al., 2017). The personal genetic information of patients could be measured by multi-omics technologies, including single-cell (SC) multi-omics. This Research Topic consists of nine manuscripts, covering a diverse range of topics from the computational analyses on various bulk and SC omics data to their applications to uncover underlining disease mechanisms, identify optimal diagnostic and prognostic biomarkers, and discover therapeutic targets and corresponding drugs for individual patients.

The review *RNA-seq data science: From raw data to effective interpretation* (Deshpande et al.) introduced basic concepts of RNA-seq data and defined discipline-specific jargon. Various RNA-seq technologies and their advantages as well as limitations were discussed in this review. Moreover, it described the major steps of computational analysis of RNA-seq data, beginning from the processing of raw data to the uncovering of biological insights, which is helpful to explore the mechanism of disease and thus identify related biomarkers at the molecular level.

Understanding the biomarkers of diseases is vital in identifying cell diversity and molecular classification, and single-cell RNA-seq (scRNA-seq) data is an effective tool for this purpose. Zhao et al. analyzed scRNA-seq data from 23 colon cancer patients to discover biomarker genes for various cancer-associated fibroblast (CAF) subtypes. This helped classify colon cancer patients into six groups and provided new insights into the significant role of CAF in cancer treatment. The CAF-related signature genes were also utilized to develop a prognostic model for colon cancer patients using LASSO Cox regression. The model surpassed traditional clinical feature-based models in predicting the prognosis of colon cancer patients. It is worth noting that other researchers have also developed prognostic models for pancreatic cancer (Tao et al.), hepatocellular carcinoma (Liu et al.), and acute myeloid leukemia (Shi et al.) using a similar bioinformatic framework.

Apart from molecular classification and prognostic model construction, multi-omics analyses were often conducted to investigate the biological functions of biomarkers or therapeutic target genes. Zhu et al. studied the complex functions of REV1, a member of the translesion synthesis DNA polymerase Y family, at multi-omics levels. The authors combined single nucleotide polymorphisms (SNPs), gene expression, and drug sensitivity information to explore the role of REV1 in carcinogenesis and prognosis as well as predicting drug sensitivities of specific signaling pathways. The study demonstrated that REV1 had the potential to be a novel prognostic biomarker for various cancers.

Genes play a vital role in various biological processes, with both protein-coding (mRNAs) and non-coding (ncRNAs) genes being integral components. Among the latter, microRNAs (miRNAs) are short endogenous RNAs consisting of 20–25 nucleotides (Ambros, 2001). They regulate gene expression post-transcription and are currently being studied for their potential to serve as biomarkers for premature ovarian failure (POF). Zhang et al. have identified miRNA-190a-5p as a promising biomarker after conducting bioinformatics analysis with miRBase and TargetScan, as well as animal experimental validation. In rats, this miRNA activates primordial follicles by targeting the expression of PHLPP1 and key proteins in the AKT-FOXO3a and AKT-LH/LHR pathways. These findings highlight miRNA-190a-5p’s potential as a therapeutic target for POF and call for further research in this area.

Screening or designing optimal drugs based on biomarkers and targets is a significant step in drug discovery. Zhang et al. developed a deep learning framework that uses convolutional neural networks (CNNs) and attention mechanisms to predict drug-protein interactions (DPIs). Attention mechanisms help identify relevant information features and improve DPI prediction performance. Guan et al. created a computational model, BNEMDI, to identify miRNA-drug interactions (MDIs) through drug substructure fingerprint, miRNA sequence, and MDIs bipartite graph. BNEMDI is stable and effective, as demonstrated by identifying miRNAs that potentially interact with 5-fluorouracil (5-FU). Understanding drug-miRNA relationships is crucial for investigating drug function mechanisms and developing treatments.

In summary, these manuscripts involved reviews, new computational pipelines, and new models with multi-omics data, helping to enhance biological mechanism study, drug discovery, and individualized medicine. Altogether, they provide a broad overview of the current status of biological and pharmaceutical integrative analysis, showing promising advances in multi-omics-based research through the application of computational approaches.
